# Does nitrogen fertilization history affects short-term microbial responses and chemical properties of soils submitted to different glyphosate concentrations?

**DOI:** 10.1371/journal.pone.0178342

**Published:** 2017-05-26

**Authors:** Elodie Nivelle, Julien Verzeaux, Amélie Chabot, David Roger, Fabien Spicher, Jérôme Lacoux, Jose-Edmundo Nava-Saucedo, Manuella Catterou, Thierry Tétu

**Affiliations:** 1 Ecologie et Dynamique des Systèmes Anthropisés (EDYSAN, FRE 3498 CNRS UPJV), Laboratoire d’Agroécologie, Ecophysiologie et Biologie Intégrative, Université de Picardie Jules Verne, 33 rue St Leu, Amiens, France; 2 Département Sciences de la Vie et de la Terre, Université de Picardie Jules Verne, 33 rue St Leu, Amiens, France; University of Porto, PORTUGAL

## Abstract

The use of nitrogen (N) fertilizer and glyphosate-based herbicides is increasing worldwide, with agriculture holding the largest market share. The agronomic and socioeconomic utilities of glyphosate are well established; however, our knowledge of the potential effects of glyphosate applied in the presence or absence of long-term N fertilization on microbial functional activities and the availability of soil nutrients remains limited. Using an ex situ approach with soils that did (N+) or did not (N0) receive synthetic N fertilization for 6 years, we assessed the impact of different rates (no glyphosate, CK; field rate, FR; 100 × field rate, 100FR) of glyphosate application on biological and chemical parameters. We observed that, after immediate application (1 day), the highest dose of glyphosate (100FR) negatively affected the alkaline phosphatase (AlP) activity in soils without N fertilization history and decreased the cation exchange capacity (CEC) in N0 compared to CK and FR treatments with N+. Conversely, the 100FR application increased nitrate (NO_3_^-^) and available phosphorus (PO_4_^3-^) regardless of N fertilization history. Then, after 8 and 15 days, the N+\100FR and N+\FR treatments exhibited the lowest values for dehydrogenase (DH) and AlP activities, respectively, while urease (URE) activity was mainly affected by N fertilization. After 15 days and irrespective of N fertilization history, the FR glyphosate application negatively affected the degradation of carbon substrates by microbial communities (expressed as the average well color development, AWCD). By contrast, the 100FR treatment positively affected AWCD, increasing PO_4_^3-^ by 5 and 16% and NO_3_^-^ by 126 and 119% in the N+ and N0 treatments, respectively. In addition, the 100FR treatment resulted in an increase in the average net nitrification rate. Principal component analysis revealed that the 100FR glyphosate treatment selected microbial communities that were able to metabolize amine substrates. Overall, the lack of N fertilization in the 6 past years combined with the highest glyphosate application rate (100FR) induced the highest values of AWCD, functional diversity, NO_3_^-^, PO_4_^3-^ and nitrification. We concluded that the intensive use of N fertilization for 6 years may change the non-target effects of glyphosate application on enzyme activities. The functional activities, nitrification and nutrient contents were increased by glyphosate only when applied at 100 times the field application rate.

## 1. Introduction

Recently, studies revealed that microbial communities may provide many benefits to crops throughout their growth [1,2]. However, such potential agroecosystem services can be disturbed by several management practices such as herbicide [3] and fertilizer [4,5] applications in intensive agriculture. Consequently, microbial responses to various soil management systems and subsequent effects on nutrient cycling are currently under investigation.

One of the most studied cultural practices is nitrogen (N) fertilization, which has been shown to induce several shifts in microbial community composition and activity over short [6–9] and long-term periods [10–12]. In general, N fertilization may reduce pH and urease, dehydrogenase and phosphatase activities, as well as microbial functional [13] and taxonomic diversity [9,11,12]. More precisely, ammonia-oxidizing archaea and bacteria can be negatively affected by N fertilization [14]. However, the consequences on N-cycling processes remain unclear since N fertilization has been reported to increase [12] and decrease [13] nitrification capacity. Such potential effects on nitrification are thus confusing since increased microbial activity may be beneficial (through greater availability of nutrients) or detrimental (through competition for nutrients with microbial populations) for plant growth [15]. Another crop management practice broadly studied is the use of herbicides, among which glyphosate is the most widespread herbicide worldwide [16]. The high popularity of glyphosate is related more to its selective utilization in transgenic glyphosate-resistant crops [17,18] than to its use as a complete herbicide between two main crops (e.g., in Europe where transgenic plants are not authorized). However, it is well known that glyphosate applications may increase yields by up to 30% in many European systems [19]. Furthermore, its replacement by plowing and other mechanical tillage operations is unfavorable for conserving soil microbial functional activity [8,20,21] or the greenhouse gases emissions budget [22]. However, the use of glyphosate for weed control in intensive agriculture is currently under debate due to its potential consequences on human health [23,24], but it has also been revealed that this herbicide may induce several non-target effects on earthworms [25] and the activities of several enzymes [26]. Overall, the potential impacts of glyphosate on soil microbiota and microbial processes remain unclear, with contrasting results. Indeed, glyphosate has been shown to induce minor or no effects on soil respiration, microbial biomass, and microbial community structure and functions, even when applied at higher doses than recommended field rates [27–31]. In parallel, stimulatory effects of microbial functional responses following glyphosate application were reported by Zabaloy et al. [29], Gomez et al. [30], Haney et al. [32] and Lane et al. [33].

Nevertheless, to our knowledge, the interaction between soil nitrogen management history and glyphosate applications has never been studied. To fill this knowledge gap, a laboratory experiment was designed to subject soil cores to three rates of glyphosate: a field rate (FR) to simulate the normal agricultural use of glyphosate, 100 times the field rate (100FR) to simulate the chemical spills that occur frequently in the fields or urban green spaces and a control (CK) without glyphosate. These three doses were chosen to determine if the microbial and chemical responses of soils treated with the field rate of glyphosate was closer to untreated or to heavily contaminated soils. Soil was sampled in a designated field station where the soil had been subjected to a 6-year crop rotation with (N+) or without (N0) N fertilizer. Soil microbial activities, functional diversity, nitrification, and nutrient availability were measured three times (1, 8 and 15 days (d)) following glyphosate application.

## 2. Materials and methods

### 2.1. Soil sampling

Sampling was conducted in November 2015 within the experimental site “La Woestyne” in Northern France (50°44’N, 2°22’E, 40 m above sea level). The owner of the land “Bonduelle company” gave permission to conduct the study on this site. The field studies did not involve endangered or protected species. Prior to 2010, the field was prepared using a chisel plough and rotary power system, fertilized conventionally, and cultivated with wheat (*Triticum aestivum*). In 2010, winter cover crops were seeded directly, and the experimental field was split into two N fertilization regimes (without (N0) or with (N+) N fertilizer). The crop rotation included green pea (*Pisum sativum* L.), maize (*Zea mays* L.), wheat (*Triticum aestivum* L.), flax (*Linum usitatissimum* L.), beet (*Beta vulgaris* L.) and wheat. The N0 plot measured 7 × 8 m while the N+ plot measured 14 × 8 m. A 7-m wide corridor separated the N0 and N+ plots to avoid N contamination. The N+ regime was determined according to the N budget method [34], and the fertilizer consisted of 50% urea, 25% ammonia and 25% nitrate. Since 2010, 650 kg N ha^-1^ have been added to the N+ plot, and the N0 plot has not been fertilized. Neither potassium-phosphate nor other elements were applied throughout the field experiment. The soil is classified as a silty clay loam with the following properties: 66% silt, 22% clay, 11% sand, 22.5 g kg^-1^ organic matter, 0.13 g kg^-1^ available phosphorus, 13.1 g kg^-1^ organic C, 1.48 g kg^-1^ total N, 11.7 cmol kg^-1^ of cation exchange capacity and a pH of 6.28. Fifty 20-cm depth soil cores were sampled in each plot by using a 7-cm diameter auger. Soils were then mixed and sieved through a 2-mm mesh. A sub-section was then separated for chemical analyses, which were performed before beginning the laboratory experiment (T0).

### 2.2. Establishment of the laboratory incubation

A 15-day laboratory incubation was carried out to assess the effects of glyphosate application on the dynamics of chemical elements and microbial activities in soil without nitrogen fertilizer (N0) and in soil to which nitrogen fertilizer was applied over a period of 6 years (N+). Six replicate pots were used for each treatment combination (N0\CK, N+\CK, N0\FR, N+\FR, N0\100FR, N+\100FR), and all of them were completely randomized. For the incubation experiment, 1600 g of fresh homogenized soil at 75–80% water holding capacity (WHC) were placed into plastic pots (2.2 L). Glyphosate, in the isopropylamine salt form, was then added on plant-free soils at the following rates: control (CK) and two different doses: 0.96 mg active ingredient per kg^-1^ soil dry weight (a.i./dw; FR) and 96 mg kg^-1^ soil (a.i/dw; 100FR), prepared with deionized water. FR corresponds to the conventional recommended field application dose (720 g active ingredient ha^-1^, [28, 30]) for soil with a bulk density of 1.45 g cm^-3^. The 100FR treatment corresponds to 100 times the recommended field rate. CK received equal to the amount of deionized water added to the glyphosate treatments. The pots were incubated in a dark room at 22 ± 1°C for 15 days. Throughout the incubation period, the water content was held constant through weekly water addition. After 1, 8 and 15 days of incubation, 150 g of fresh samples were collected from the pots and divided into two parts: the first part was stored at 4°C for Biolog® and chemical analyses, and the second part was stored at -20°C for enzyme activity measurements. The moisture content, pH, cation exchange capacity (CEC), total organic carbon (TOC), total nitrogen (TN), extractable nitrates (NO_3_^-^), available phosphorus (PO_4_^3-^), microbial enzyme activities (dehydrogenase (DH), alkaline phosphatase (AlP) and urease (URE)) and Community Level Physiological Profiles (CLPP) were measured.

### 2.3. Microbial analyses

#### 2.3.1. Determination of enzyme activities

Dehydrogenase activity (DH) based on the reduction of the 2,3,5-triphenyltetrazoliumchloride (TTC) to triphenyl tetrazolium formazan (TPF) was determined according to Casida [35] with some modifications [8]. Soil samples prepared with CaCO_3_ (100: 1 fresh mass ratio) were mixed both in a 2,3,5-triphenyltetrazoliumchloride (TTC) solution (3%) and in a deionized water and then incubated at 37°C for 24 h in a dark room. After incubation, TPF was extracted with a solution of pure methanol. After filtration, TPF concentrations were determined by spectrophotometry at 485 nm. Alkaline phosphatase activity (AlP) was determined according to the method described by Tabatabai and Bremner [36]. Fresh soil samples were pre-incubated at 27°C for 48 h. After pre-incubation, deionized water was added to soil samples (1:1 mass ratio) and mixed. A part of soil samples was used and substrate *p*- nitrophenyl phosphate solution (pNPP, 1%) and borate buffer (pH 9) were added. After incubation at 37°C for 1 h, CaCl_2_ (0.5 N) and NaOH (1 N) were added to stop the reaction. Finally, all samples were centrifuged at 10 000 *g* for 5 min. The released *p*-nitrophenol (*p*NP) was measured at 405 nm. Urease activity (URE) was determined using the method of Alef and Nannipieri [37]. A mixture of fresh soil with urea solution (79.9 mM) (1:0.5 fresh mass ratio) was incubated at 37°C for 2 h before adding KCl (2.0 M). After 30 min of agitation, the suspension was filtered. To 1 ml of filtrate were added 9 ml deionized water, 2 ml sodium dichloroisocyanurate (0.1%) and 5 ml of a mixture of 1.06 M sodium salicylate/ 0.3 M sodium hydroxide/deionized water (1: 3/ 1:3/ 1: 3, vol/ vol/ vol). After decanting for 30 min at room temperature and in a dark chamber, ammonium was measured at 690 nm.

#### 2.3.2. Community level of physiological profiles

CLPP were assessed using Biolog EcoPlates^TM^ (BIOLOG, Hayward, USA) as described by Govaerts et al. [38]. The analysis of CLPP was completed within 24 hours after sampling. Briefly, 10 g of fresh soil samples were shaken for 60 min with 90 ml of sterilized saline solution (0.85% NaCl, w/v) and brought to a 10^−3^ final dilution. Each well of the Biolog EcoPlates was inoculated with 150-μl in two replicates by extract. The plates were incubated at 25°C in the dark for 196 h and read at 590 nm every 24 h. Data recorded at the exponential phase (120 h) were used to calculate the average well color development (*AWCD*) following Eq (1) and Shannon index (*H’*) following Eq (2). *AWCD* and *H’* represent the soil catabolic potential and diversity respectively.

$$AWCD=\sum OD_{i}/31$$ (1)

Where, *OD*_*i*_ is the optical density value from each well after subtracting both the value at day 0, to eliminate background color generated by substrates and the bacterial suspension, and the value of the blank (water).

$$H’=\text{-}\sum P_{i}\;x\;\ln\left(P_{i}\right)$$ (2)

Where, *P*_*i*_ is the ratio of the activity on each substrate (*OD*_*i*_) to the sum of activities on all substrates *∑OD*_*i*_ using an optical density (*OD*) of 0.25 as threshold for positive responses [39].

### 2.4. Chemical analyses

Moisture was measured after drying samples at 105°C for 24 h. The pH measurements were performed after shaking the dried soil with deionized water (1:5 dry mass ratio) for 45 min. The mixture was then left for 5 min and the pH recorded using a pH meter FE20-FiveEasy^TM^ (Mettler Toledo, Switzerland). CEC was quantified by shaking the soil samples for 1 h with a solution of cobaltihexamine chloride (1.66 mol L^-1^) (1:10 mass ratio) [40]. After filtration, optic density was measured at two wavelengths (475 and 380 nm) using an Eon spectrophotometer (BioTek Instruments Inc., USA). For TOC, TN and PO_4_^3-^, sieved soil was oven-dried at 45°C for 48 h and finely ground by using a ball-mill (Retsch, MM400). TOC and TN were analyzed using a CN elemental analyzer (Flash EA 1112, Thermo Electron, Germany). Since analyses performed before the experiment setup revealed that the soil was free of carbonate, the soil total C was assumed to be equal to the TOC. The NO_3_^-^ content of the soils was determined by extracting the soil samples with 2 M KCl (1:5 fresh mass ratio) for 1 h on a rotary shaker. The extracts were centrifuged 10 min at 1800 x g and supernatants were analyzed by continuous flow analytical system (Alpkem flow solution IV power base, OI Analytical, USA). Taking the NO_3_-N contents (NO_3_^-^) before the incubation (T0) as baselines, the net nitrification rate (*NIT*) was calculated as fallows (Eq 3):

NIT=(Nt-N0)/t (3)

Where, *N0* and *Nt* are the NO3-N (including NO2-N) contents in the soil at time 0 and *t* days after incubation [41].

The PO_4_^3-^ content was determined by extracting the soil samples with 0.5 M NaHCO_3_ pH 8.5 (1:20 dry mass ratio) and was measured in the extract by colorimetric method at 882 nm [42].

### 2.5. Data analysis

All statistical analyses were performed using R software v. 3.1.2 (R Development Core Team, http://www.R-project.org, 2014). In figures and tables, differences between treatments at each incubation time were performed by one-way analysis of variance (ANOVA) and a post-hoc Tukey HSD-test was used to determine the significant differences among treatments (*p*< 0.05). ANOVA and Tuckey tests were performed by using the agricolae package [43]. When Bartlett and Shapiro tests revealed a lack of homoscedasticity or normality of a data set, a non-parametric Kruskall-Wallis test followed by a Conover post-hoc test (*p*< 0.05) were performed by using the PMCMR package [44]. Values given in figures and tables correspond to the average of 6 data (n = 6) ± standard error (S.E).

Soil chemical and microbial properties data were analyzed by using a two-way ANOVA with (i) incubation times or fertilizer-N application and (ii) herbicide application as factors. Principal Component Analyses (PCA) were performed using vegan package [45]. For statistical analyses, the 31 substrates of Biolog EcoPlates were grouped into six substrate groups, (a) phosphate carbon, (b) amines, (c) amino acids, (d) polymers, (e) carboxylic acids, and (f) carbohydrates. These groups of carbon sources data were subjected to PCA to reduce complex multidimensional data and allow a more straightforward interpretation of results. A second PCA was performed to establish relationships among soils subjected to different treatments and multiple chemical and biological variables analyzed.

## 3. Results

### 3.1. Effects of N fertilization and glyphosate on soil enzyme activities

**Fig 1:**
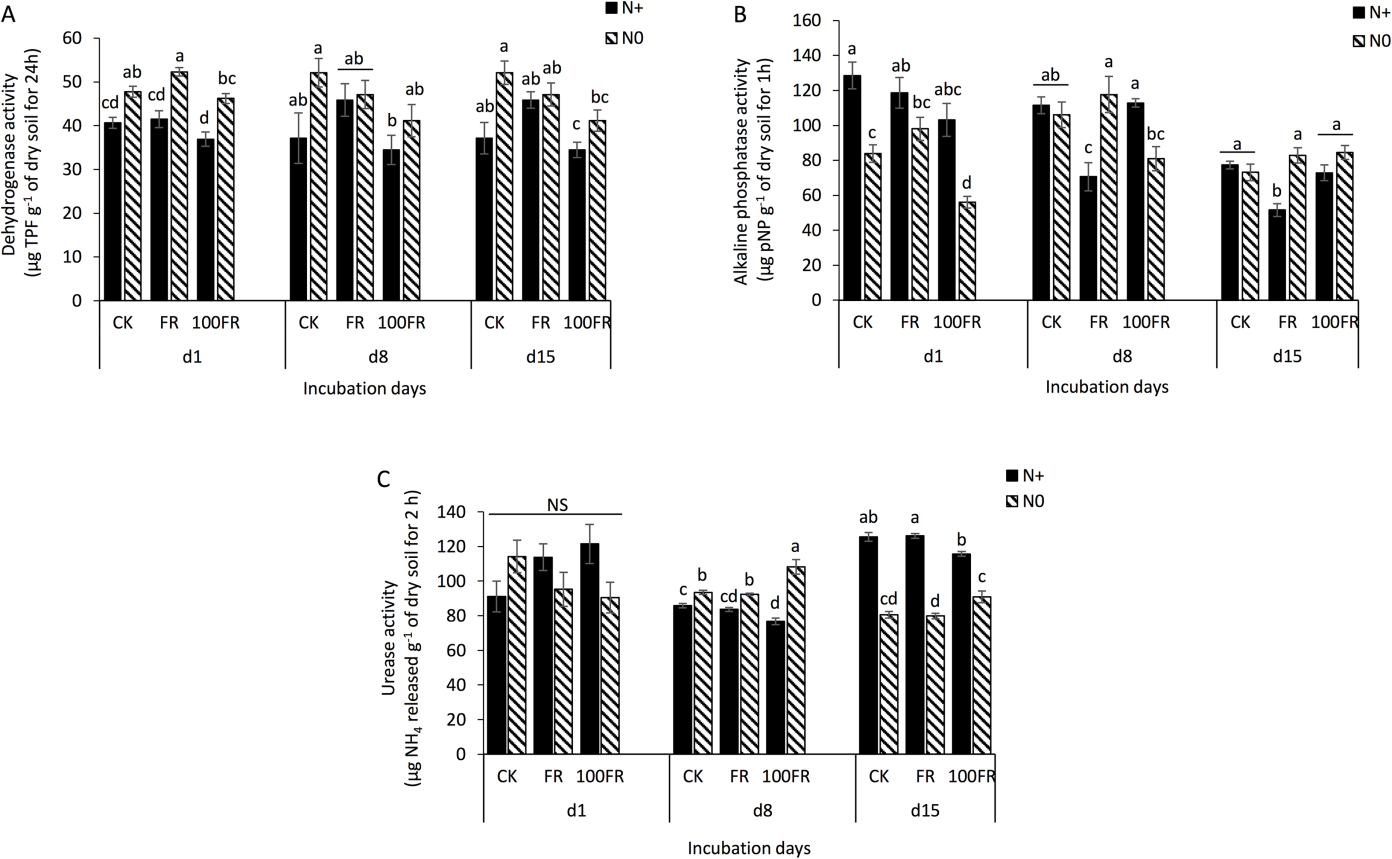
**Enzyme activities of (A) dehydrogenase, (B) alkaline phosphatase and (C) urease in glyphosate-free and glyphosate-treated soils under two N fertilization histories.** Different letters denote significant differences according to Tukey HSD test (*P* < 0.05) among all fertilization and herbicide application treatments at each incubation time (1, 8, 15 days). CK: glyphosate-free, FR: field rate, 100FR: 100 × field rate, N+: N-fertilized for 6 years, N0: unfertilized for 6 years.

On the first day following glyphosate application, the highest dose (100FR; 100 times the conventional field application rate) decreased alkaline phosphatase (AlP) activity (d1, 100FR, Fig 1B) in unfertilized soil (N0), but no effects were observed for dehydrogenase (DH), and urease (URE) (Fig 1A and 1C). In parallel, soils that received N fertilization for 6 years before the beginning of the experiment exhibited lower DH (black histograms, Fig 1A) and higher AlP (black histograms, Fig 1B) values compared to unfertilized soils, irrespective of the herbicide rate.

After 8 days, the DH and AlP activities in the unfertilized soils that received the FR and 100FR glyphosate application did not differ from the control (CK), while in the fertilized soils, AlP decreased significantly in response to the FR application (Fig 1A and 1B). URE activity increased in unfertilized soils and decreased in fertilized soils in response to the 100FR application compared with CK. The unfertilized soils had significantly higher URE values than fertilized soils, irrespective of glyphosate application (Fig 1C).

After 15 days, the DH activity was similar to that measured at 8 days (Fig 1A), and the AlP values were significantly lower in the FR treatment with N fertilization compared to all other treatments (Fig 1B) revealing a combined effect of nitrogen management history and glyphosate application. The URE activity was not affected by glyphosate application but increased significantly in fertilized soils compared to unfertilized soils, irrespective of the glyphosate application rate (Fig 1C).

### 3.2. Effect of N fertilization and glyphosate on C source utilization

**Fig 2:**
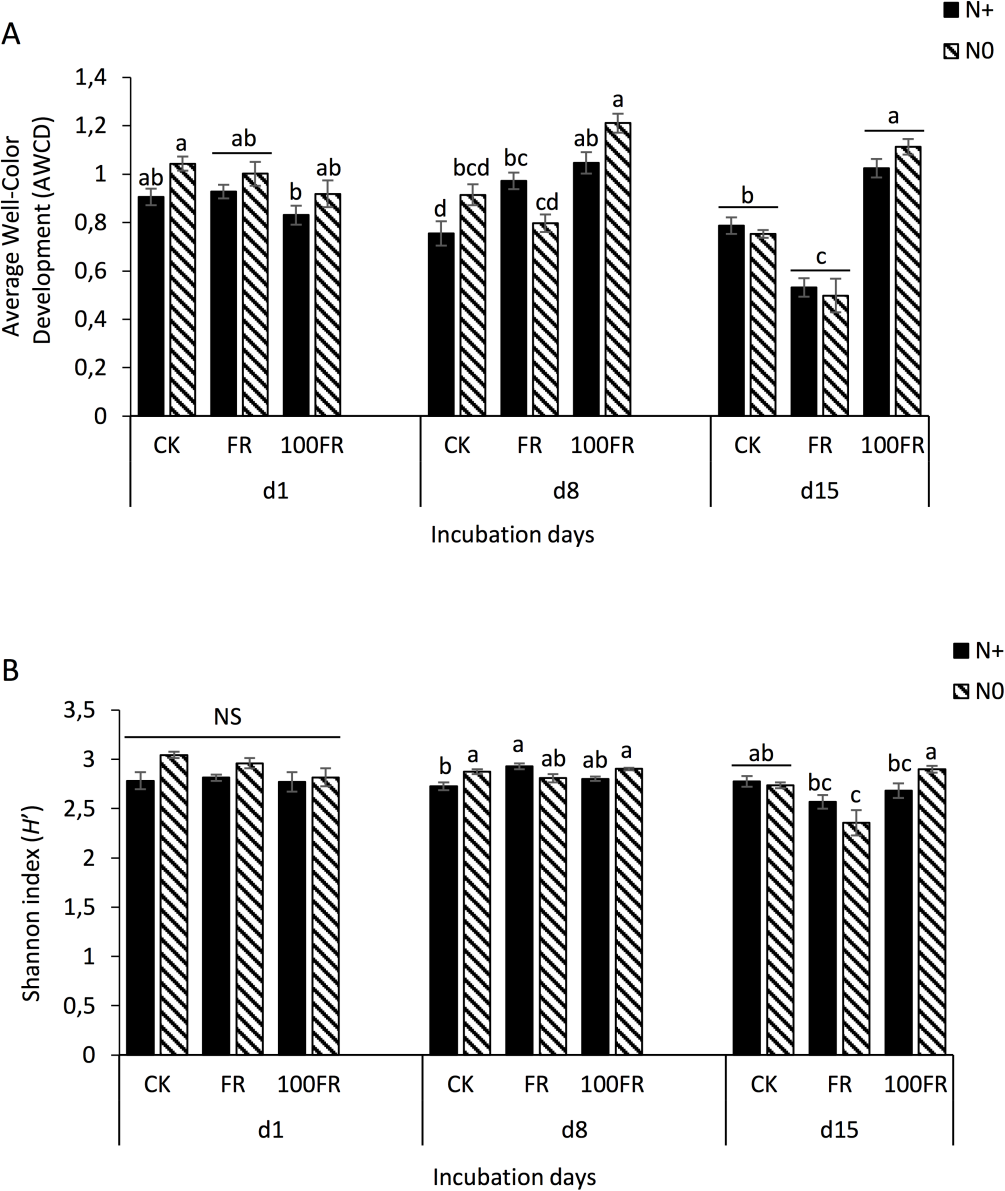
**Values of (A) average well color development and (B) Shannon diversity index *H*’ of culturable microbial communities on BIOLOG ecoplates of glyphosate-free and glyphosate-treated soils under two N fertilization histories.** Different letters denote significant differences according to Tukey HSD test (*P* < 0.05) among all fertilization and herbicide treatments at each incubation time (1, 8, 15 days). CK: glyphosate-free, FR: field rate, 100FR: 100 × field rate, N+: N-fertilized for 6 years, N0: unfertilized for 6 years.

After 1 day, no effect was observed on the microbial functional activities (AWCD) or the microbial functional diversity (Shannon index, *H*’) in the N0 and N+ soils (Fig 2A and 2B, respectively) except for a decrease in the AWCD in the fertilized soil that received the 100FR application compared with the unfertilized and glyphosate-untreated soil (Fig 2A). After 8 days of incubation, glyphosate applications (FR and 100FR) significantly increased the AWCD in N+ soils compared to untreated soil (CK), while only the high glyphosate rate (100FR) significantly increased the AWCD in N0 soils. The highest AWCD values were measured in the unfertilized (N0) soil that received the 100FR glyphosate application (Fig 2A). In N+ soils, the lack of glyphosate (CK) resulted in the lowest values for both microbial functional activity (AWCD) and diversity (*H*') (Fig 2A and 2B).

Conversely, 15 days following herbicide application, the FR application led to lower values of AWCD in both N+ and N0 soils (Fig 2A) and had a negative effect on the Shannon diversity index (*H*’) of the N0 soils. The diversity index did not differ significantly between the untreated and 100FR-treated soils. We also noted that the diversity index was higher in N0 soils following the 100FR glyphosate application compared with the other treatments except the control (CK) (Fig 2B).

**Fig 3:**
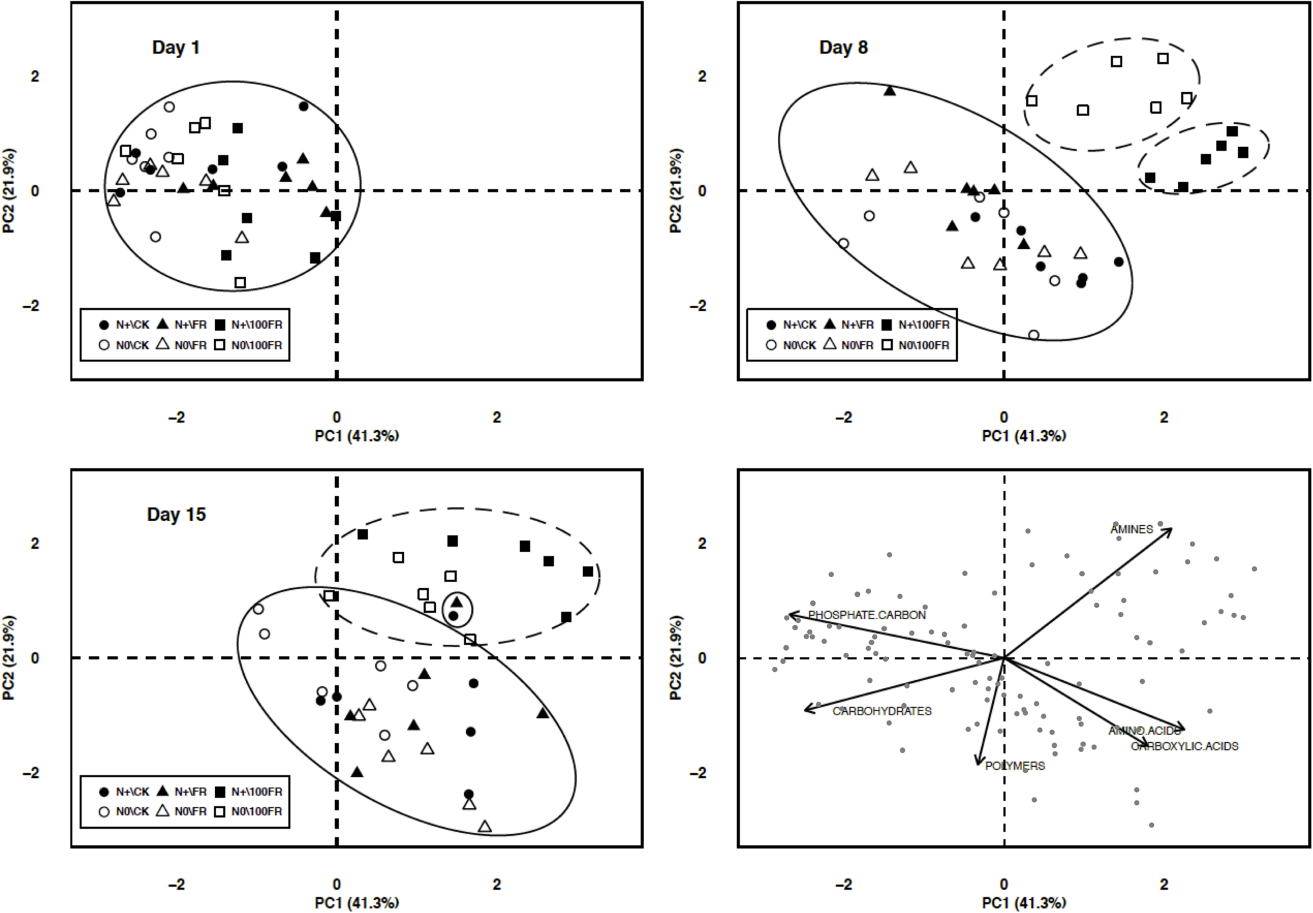
Principal component analysis based on the data of the carbon substrates groups from Biolog EcoPlates at each incubation time (1, 8, 15 days). N+\CK: fertilized soils and glyphosate-free (black circles), N+\FR: fertilized soils and field application rate of glyphosate (black triangles), N+\100FR: fertilized soils and 100 times the field application rate of glyphosate (black squares), N0\CK: unfertilized soils and glyphosate-free (white circles), N0\FR: unfertilized soils and field application rate of glyphosate (white triangles), N0\100FR: unfertilized soils and 100 times the field application rate of glyphosate (white squares).

Principal component analysis (PCA) was conducted using the 6 groups in which the 31 carbon substrates of Biolog EcoPlates^TM^ (Biolog Inc., Hayward, CA, USA) were grouped (Fig 3). The first PCA axis (PC1, proportion of variance explained: 41.3%) was characterized by a gradient of increasing amino/carboxylic acids and decreasing carbohydrates and phosphate carbon. The second axis (PC2, 21.9%) was characterized by a gradient of increasing amines and decreasing polymers. It should be noted that initially (day 1), the different treatments were combined and correlated with axis 1 (negative scores). The microbial communities that were present in these soils preferentially degraded the carbohydrates and phosphate-carbon sources. From day 8, a differentiation of the microbial communities was observed between the treatments. The 100FR glyphosate application generated positive scores for N0 soils on axis 1 and for N+ soils on axis 2. Generally, the addition of high concentration of glyphosate to soils fertilized or no with nitrogen correlated with amine-degrading microbial communities in these soils. In soils that received synthetic N for 6-years but no glyphosate, a positive correlation was observed for microbial communities that degrade amino acids and carboxylic acids. After 15 days of incubation, the microbial communities in the N+ soil with a high concentration of glyphosate degraded more amines, similar to the microbial communities in the N0 soils. By contrast, after 8 days, the microbial communities in the untreated CK and FR soils preferentially degraded polymers (mainly negative scores on axis 1). After 15 days, these communities preferentially degraded polymers (negative scores on axis 1) and amino and carboxylic acids (positive scores on axis 2). The low dose of glyphosate (FR) did not induce changes in the substrate degradation pattern by microorganisms compared with untreated soil (CK), irrespective of the N fertilization history.

### 3.3. Effects of N fertilization and glyphosate on soil chemical parameters

Table 1 reports the pH, soil TOC, TN, C:N, and CEC values of soils from the different treatments. Significant differences were observed on the first day following glyphosate application for TOC and CEC. Although no effect related to glyphosate treatment was observed between the N0 and N+ soils, we noted significantly higher TOC values for N0 in the glyphosate-treated soils (FR and 100FR) compared to untreated N+ soil (CK). Conversely, a significant decrease in CEC was observed for the N0 soil treated with 100FR glyphosate compared with untreated N+ soil (CK) or soil that received the FR glyphosate application.

**Table 1:** Soil chemical properties measured at each incubation time (1, 8, 15 days) under two N fertilization histories and three doses of glyphosate application.

Parameter	N fertilization	Glyphosate dose	Incubation time (days)		
			1	8	15
Moisture (%)	N+	CK	17.89 ± 0.24	18.34 ± 0.78	17.21 ± 0.33
		FR	18.60 ± 0.42	17.82 ± 0.24	17.80 ± 0.23
		100FR	18.28 ± 0.12	17.38 ± 0.13	17.70 ± 0.17
	N0	CK	19.11 ± 0.61	17.03 ± 1.16	17.83 ± 0.16
		FR	18.66 ± 0.24	18.48 ± 0.21	18.12 ± 0.17
		100FR	18.34 ± 0.15	18.50 ± 0.18	17.59 ± 0.24
pH	N+	CK	6.81 ± 0.07	6.89 ± 0.05	6.95 ± 0.05
		FR	6.91 ± 0.05	6.88 ± 0.04	6.93 ± 0.01
		100FR	6.93 ± 0.03	6.30 ± 0.02	6.91 ± 0.02
	N0	CK	6.82 ± 0.07	6.89 ± 0.03	6.96 ± 0.03
		FR	6.87 ± 0.08	6.88 ± 0.04	6.98 ± 0.02
		100FR	6.86 ± 0.05	6.83 ± 0.03	6.97 ± 0.03
TOC (mg kg^-1^)	N+	CK	11.33 ± 0.28^b^	12.05 ± 0.22	12.83 ± 0.13
		FR	11.00 ± 0.30^ab^	12.81 ± 0.63	13.00 ± 0.35
		100FR	11.84 ± 0.22^ab^	13.65 ± 1.23	12.74 ± 0.28
	N0	CK	12.19 ± 0.17^ab^	12.94 ± 0.61	13.26 ± 0.28
		FR	12.71 ± 0.47^a^	12.74 ± 0.23	13.61 ± 0.33
		100FR	12.76 ± 0.38^a^	12.43 ± 0.29	13.35 ± 0.28
TN (mg kg^-1^)	N+	CK	1.17 ± 0.03	1.24 ± 0.03	1.33 ± 0.03
		FR	1.22 ± 0.03	1.25 ± 0.02	1.39 ± 0.02
		100FR	1.25 ± 0.07	1.21 ± 0.03	1.31 ± 0.02
	N0	CK	1.18 ± 0.02	1.17 ± 0.01	1.38 ± 0.04
		FR	1.20 ± 0.02	1.22 ± 0.03	1.42 ± 0.02
		100FR	1.27 ± 0.07	1.20 ± 0.02	1.44 ± 0.05
Soil C:N	N+	CK	9.68 ± 0.12	9.91 ± 0.14	9.63 ± 0.22
		FR	9.87 ± 0.24	10.26 ± 0.49	9.39 ± 0.32
		100FR	9.57 ± 0.30	11.23 ± 0.78	9.73 ± 0.07
	N0	CK	10.32 ± 0.11	11.02 ± 0.47	9.63 ± 0.12
		FR	10.60 ± 0.43	10.42 ± 0.11	9.59 ± 0.11
		100FR	10.10 ± 0.39	10.36 ± 0.21	9.32 ± 0.28
CEC (cmol kg^-1^)	N+	CK	14.20 ± 0.43^a^	11.08 ± 0.17	11.29 ± 0.32
		FR	13.05 ± 0.72^a^	10.67 ± 0.34	11.16 ± 0.41
		100FR	12.14 ± 0.77^ab^	10.91 ± 0.24	11.49 ± 0.32
	N0	CK	12.66 ± 0.44^ab^	10.54 ± 0.30	10.48 ± 0.43
		FR	12.64 ± 0.39^ab^	11.15 ± 0.30	10.45 ± 0.29
		100FR	10.44 ± 0.34^b^	11.37 ± 0.41	10.63 ± 0.35
NO_3_^-^ (mg kg^-1^)	N+	CK	5.50 ± 0.57^b^	7.04 ± 0.31^b^	8.23 ± 0.75^b^
		FR	6.29 ± 0.30^b^	7.16 ± 0.75^b^	8.34 ± 0.22^b^
		100FR	7.21 ± 0.40^ab^	18.14 ± 1.13^a^	18.62 ± 1.14^a^
	N0	CK	5.53 ± 0.14^b^	8.23 ± 0.80^b^	10.15 ± 0.59^b^
		FR	7.35 ± 0.43^ab^	7.87 ± 0.67^b^	10.05 ± 0.19^b^
		100FR	8.50 ± 0.65^a^	18.51 ± 0.91^a^	22.27 ± 1.18^a^
PO_4_^3-^ (mg kg^-1^)	N+	CK	44.31 ± 1.09^b^	40.95 ± 1.11^b^	44.73 ± 0.65^b^
		FR	46.07 ± 0.39^b^	41.88 ± 1.51^b^	43.12 ± 0.48^b^
		100FR	51.47 ± 1.44^a^	44.40 ± 0.54^ab^	46.98 ± 0.94^a^
	N0	CK	48.24 ± 1.32^ab^	43.87 ± 1.59^ab^	45.98 ± 0.34^b^
		FR	44.47 ± 0.85^b^	44.22 ± 0.99^ab^	46.75 ± 0.43^ab^
		100FR	51.82 ± 0.59^a^	47.67 ± 1.56^a^	52.14 ± 1.41^a^

Mean values ± standard error of six replicates. Different letters indicate significant differences among treatments at *P* < 0.05. TOC: total organic carbon, TN: total nitrogen, CEC: cation exchange capacity, NO_3_^-^: nitrates, PO_4_^3-^: available phosphorus, N+: fertilized soils, N0: unfertilized soils, CK: glyphosate free, FR: recommended field rate of glyphosate application, 100FR: 100 times the field rate of glyphosate application.

During the incubation, the available phosphorus (PO_4_^3-^) contents increased by 16 and 7% after 1 day, 8 and 9% after 8 days, and 5 and 13% after 15 days in the N+ and N0 soils, respectively, in response to the 100FR glyphosate treatment compared to the untreated soil (CK) (Table 1). Overall, the 100FR glyphosate treatment applied to the N0 soil (100FR/N0) resulted in the highest PO_4_^3-^ content on day 1 compared to the other treatments, except for the N0 untreated soil (N0/CK), and on day 8 compared to the N+ soil with FR and without glyphosate (N+/FR and N+/CK). After 15 days, the 100FR glyphosate application resulted in the highest PO_4_^3-^ content in the N+ and N0 soils compared to the other treatments. Similar trends were observed for the nitrate (NO_3_^-^) content (Table 1). The nitrate content increased in the soils treated with glyphosate (FR, 100FR) compared to the untreated soil (CK) for the three incubation times. After day 1 and in comparison to the untreated soil (CK), we noted an increase in soil NO_3_^-^ of 14 and 33% for N+ and N0, respectively, in response to the FR glyphosate treatment, and of 31 and 53% for N+ and N0, respectively, in response to the 100FR glyphosate treatment. After 8 and 15 days, the values in the FR treatments were similar to those in the CK treatment. By contrast, the nitrates levels following the 100FR glyphosate application were significantly increased by 158 and 125% after 8 days and by 126 and 119% after 15 days in the N+ and N0 soils, respectively. Overall, from 1 to 8 days following glyphosate application, the 100FR glyphosate treatment significantly increased the NO_3_^-^ content compared to the other treatments.

**Fig 4:**
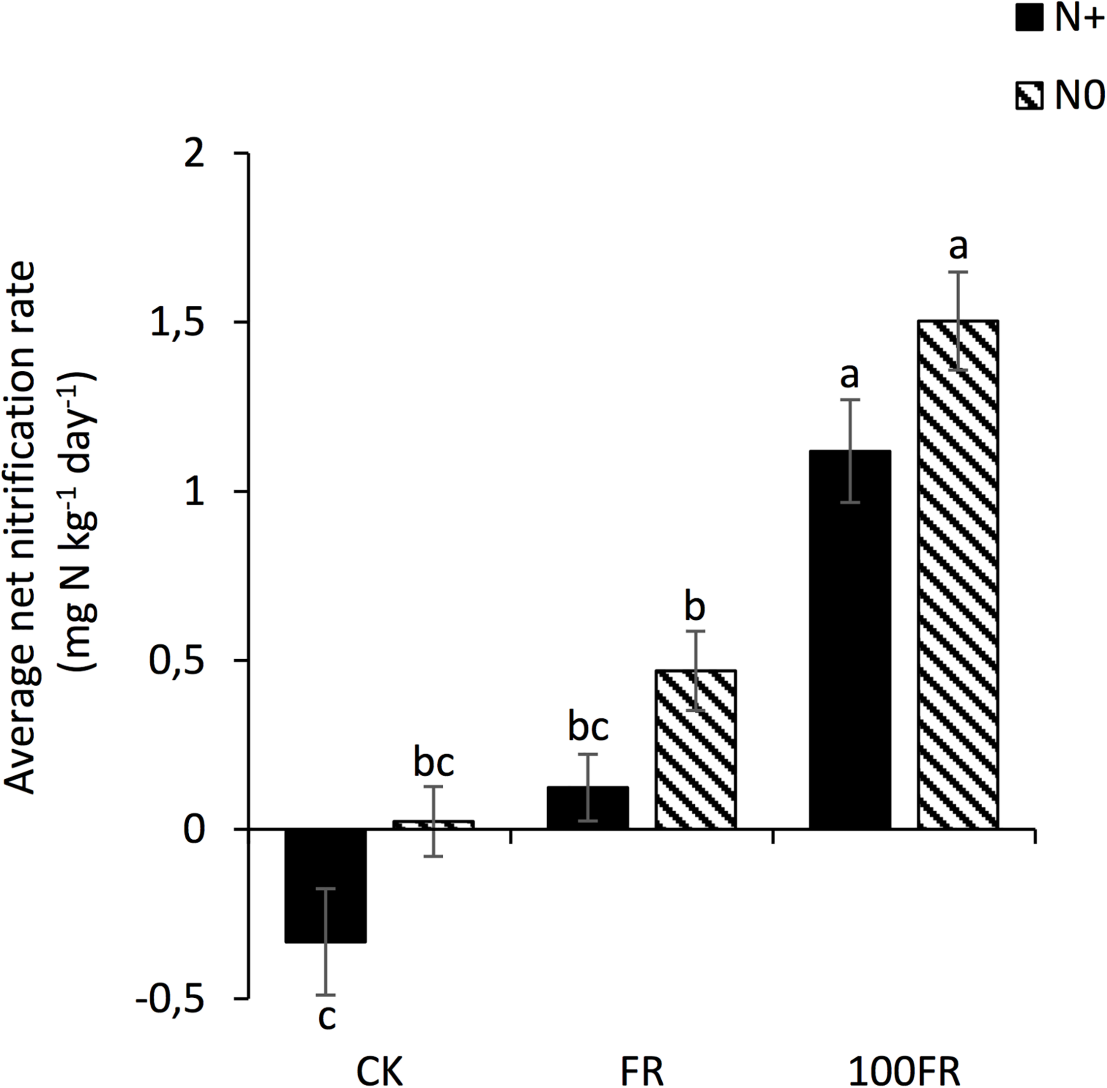
Average net nitrification rate in glyphosate-free and glyphosate-treated soils under two fertilization histories over a 15-days incubation period at 22°C. Different letters indicate a significant difference according to Tukey HSD test (*P* < 0.05). CK: glyphosate-free, FR: field rate, 100FR: 100 × field rate, N+: N-fertilized for 6 years, N0: unfertilized for 6 years.

The average net nitrification rate differed among the six soil treatments (Fig 4). Application of the glyphosate FR to unfertilized soils increased the average net nitrification rate compared to N+ soils not treated with glyphosate. The 100FR glyphosate application rate strongly increased the average net nitrification rate (which reached 1.5 mg N kg^-1^ soil in the N0 soils) compared to other treatments, irrespective of N fertilization.

### 3.4. Combined interpretation of the soil properties analyzed

Table 2 shows the significant interactions (determined using two-way ANOVA) between the herbicide rate and incubation time for NO_3_^-^, CEC, AWCD, AlP activity, and *H*’ in unfertilized soils (N0) and for NO_3_^-^, AWCD, and AlP and URE activities in fertilized soils (N+). Significant interactions between herbicide and fertilization were also found for AlP activity only. The incubation period was the factor that most strongly impacted the microbial measurements. The highest dose of glyphosate (100FR) affected NO_3_^-^, PO_4_^3-^, AWCD and DH in both fertilized and unfertilized soils and AlP activity in fertilized soils only; the N fertilization history affected PO_4_^3-^, CEC, and DH and URE activities.

**Table 2:** Analysis of variance for soil (microbial and chemical) properties as affected by herbicide (H), incubation time (T) or fertilizer (F) and their interactions (H × T or H × F).

	NO_3_^-^	PO_4_^3-^	TOC	TN	Soil C:N	CEC	DH	AlP	URE	AWCD	*H*'
**Non-fertilized** **Soils**											
H	<0.001***	<0.001***	0.307	0.137	0.985	0.079	<0.001***	0.0774	0.519	<0.001***	0.057
	*54%*	*66%*	*4*.*9%*	*6*.*1%*	*11%*	*9*.*3%*	*56%*	*36%*	*11%*	*41%*	*19%*
T	<0.001***	<0.001***	<0.001***	<0.001***	<0.001***	<0.001***	<0.001***	<0.001***	0.011*	<0.001***	<0.001***
	*30%*	*23%*	*76%*	*93%*	*81%*	*45%*	*24%*	*38%*	*47%*	*18%*	*39%*
H x T	<0.001***	0.402	0.861	0.308	0.492	<0.001***	0.318	0.015*	0.867	<0.001***	<0.001***
	*16%*	*11%*	*19%*	*0*.*7%*	*8*.*6%*	*45%*	*19%*	*26%*	*41%*	*41%*	*42%*
**Fertilized soils**											
H	<0.001***	<0.001***	0.299	0.372	0.352	0.173	0.006**	<0.001***	0.149	<0.001***	0.969
	*56%*	*44%*	*16%*	*6*.*0%*	*12%*	*8*.*0%*	*33%*	*18%*	*5*.*0%*	*23%*	*0*.*35%*
T	<0.001***	<0.001***	0.013*	<0.001***	0.008**	<0.001***	<0.001***	<0.001***	<0.001***	<0.001***	0.027*
	*24%*	*46%*	*64%*	*81%*	*59%*	*78%*	*52%*	*67%*	*78%*	*14%*	*43%*
H x T	<0.001***	0.111	0.580	0.378	0.278	0.170	0.271	<0.001***	0.022*	<0.001***	0.055
	*19%*	*10%*	*19%*	*13%*	*29%*	*14%*	*15%*	*15%*	*17%*	*62%*	*56%*
**Treatments**											
H	<0.001***	<0.001***	0.317	0.337	0.337	0.157	<0.001***	0.075	0.702	<0.001***	0.121
	*97%*	*75%*	*34%*	*58%*	*0*.*21%*	*34%*	*51%*	*22%*	*7*.*0%*	*83%*	*37%*
F	0.075	<0.001***	0.071	0.531	0.531	0.014*	<0.001***	0.164	0.018*	0.056	0.098
	*2*.*8%*	*23%*	*48%*	*11%*	*29%*	*56%*	*48%*	*8*.*2%*	*55%*	*9*.*0%*	*24%*
H x F	0.911	0.567	0.535	0.528	0.528	0.568	0.725	<0.001***	0.137	0.223	0.104
	*0*.*2%*	*1*.*7%*	*18%*	*34%*	*70%*	*10%*	*1*.*0%*	*69%*	*38%*	*8*.*0%*	*39%*

Asterisks represent significance level according to ANOVA (*P < 0.05, **P < 0.01 and ***P < 0.001).

The relative percentage of variance explained for each of the factors and their interactions are shown in italics below the *P* values. TOC: total organic carbon, TN: total nitrogen, CEC: cation exchange capacity, DH: dehydrogenase activity, AlP: alkaline phosphatase activity, URE: urease activity, AWCD: average well color development, *H*’: Shannon index.

**Fig 5:**
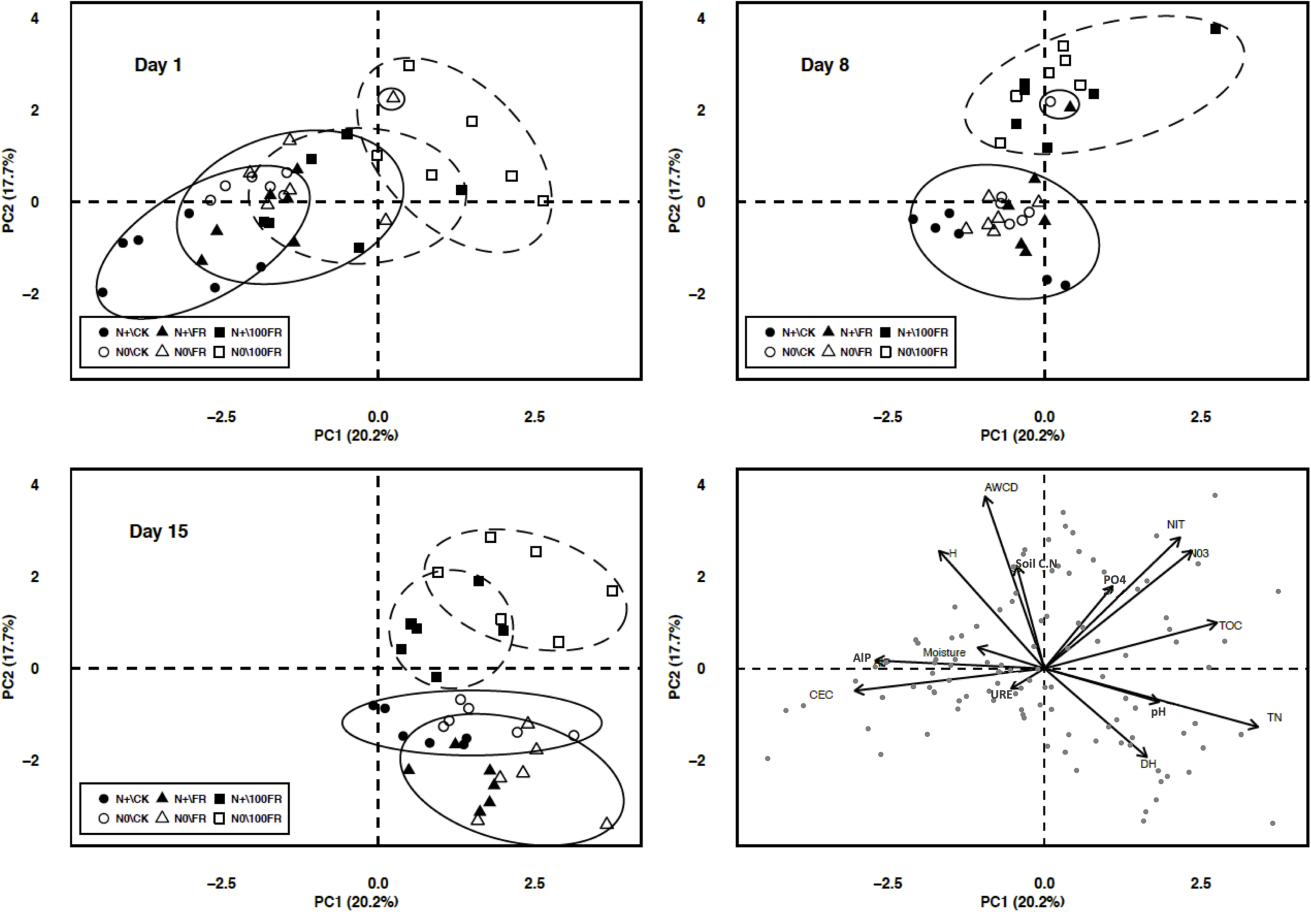
Principal component analysis based on correlations between soil microbial and chemical parameters at each incubation time (1, 8, 15 days). N+\CK: fertilized soils and glyphosate-free (black circles), N+\FR: fertilized soils and field application rate of glyphosate (black triangles), N+\100FR: fertilized soils and 100 times the field application rate of glyphosate (black squares), N0\CK: unfertilized soils and glyphosate-free (white circles), N0\FR: unfertilized soils and field application rate of glyphosate (white triangles), N0\100FR: unfertilized soils and 100 times the field application rate of glyphosate (white squares), DH: dehydrogenase activity, AlP: alkaline phosphatase activity, URE: urease activity, AWCD: average well color development, *H*’: Shannon index, CEC: cation exchange capacity, TOC: total carbon organic, TN: total nitrogen, Soil.C.N: soil C:N ratio, pH: hydrogen potential, PO_4_: available phosphorus content, NO_3_: nitrate content, NIT: average net nitrification rate, Moisture: soil moisture.

PCA, performed to highlight links between soil properties, microbial community indices and treatments, indicated that two components accounted for 37.9% of the total variance (Fig 5). The first axis (PC1, proportion of variance explained: 20.2%) was positively correlated with TOC, TN, pH and was negatively correlated with CEC, moisture, and AIP activities (Fig 5). The second axis (PC2, 17.7%) was positively correlated with AWCD, C:N and was negatively correlated with DH activity. On day 1, samples were gradually separated on PC1: the most negatively correlated with PC1 were CK followed by FR, irrespective of N fertilization history, and then the fertilized soils subjected to the 100FR treatment (soils were characterized by high values of CEC and AlP). Additionally, unfertilized soil subjected to the 100FR treatment was positively correlated with PC1 and PC2 and was characterized by high values of PO_4_^3-^, TOC, AWCD, and soil C:N. On day 8, the responses were similar, depending mainly on glyphosate concentration, regardless of N fertilization history. The CK and FR soil samples were negatively correlated with PC1 and were characterized by high values of CEC and AlP and URE activities, while the 100FR samples were positively correlated with PC2 and were characterized by high values of AWCD, *H*’, C:N, and PO_4_^3-^. Finally, on day 15, the CK and FR samples were positively correlated with PC1 and negatively correlated with PC2 and were characterized by high values of DH activity, pH and TN. On the contrary, the 100FR samples were positively correlated with both PC1 and PC2 and were characterized by high values of PO_4_^3-^, NIT, NO_3_^-^ and TOC.

## 4. Discussion

**Fig 6:**
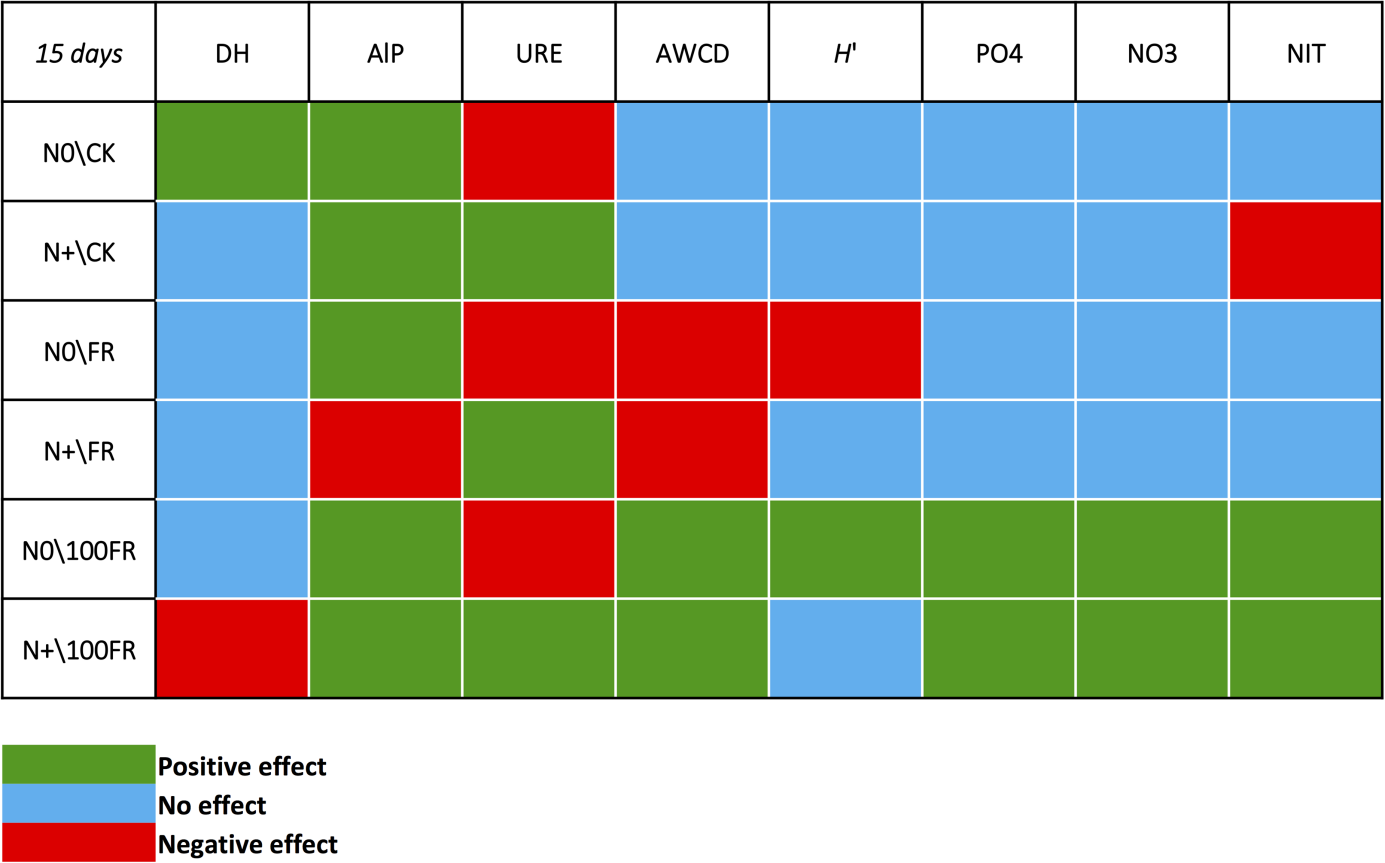
Summary of the measured effects of glyphosate application / nitrogen history combinations on soil biological and chemical parameters at 15 days following glyphosate application in the laboratory incubation. Effects of treatments on variables are coloured by green as positive, and red as negative while the absence of clear effect was coloured by blue. DH: dehydrogenase activity, AlP: alkaline phosphatase activity, URE: urease activity, AWCD: average well color development, *H*’: Shannon index, PO_4_: available phosphorus content, NO_3_: nitrate content, NIT: average net nitrification rate.

Although N fertilization is used to increase the soil inorganic N content, glyphosate is not used for this purpose. However, the latter may induce non-target effects on microbial activities, leading to increased nutrient contents. Moreover, N fertilization history may affect these non-target effects of glyphosate through previous modification of microbial communities. In the present study, we determined the effect of increasing the rate of glyphosate applied to unfertilized soil and soil that was fertilized over a 6-year period. These effects were measured through soil chemical (i.e., pH, moisture, NIT, CEC, NO_3_^-^, PO_4_^3-^, TOC, TN, soil C:N), and biological parameters involved in biogeochemical cycles (i.e., DH: overall soil biological activity involved in nutrient cycling; AlP: enzyme activity involved in the soil P cycle; URE: enzyme activity involved in soil N cycling; AWCD: metabolic potential of soils, involved in nutrients cycling, and H’: functional diversity of the soil microbial community). The study was conducted ex situ to compare the short-term effect of a control (without glyphosate) and two glyphosate rates (0.96 mg kg^-1^, 96 mg kg^-1^) applied to soils that did or did not receive N fertilizer for 6 years. To summarize the results obtained, a conceptual figure displays the effects of all the 6 combined treatments on soil biological and chemical parameters (Fig 6).

### 4.1. Nitrogen fertilization and glyphosate modify soil enzyme activities

Soil enzymes have been reported as relevant soil quality indicators and described as "biological fingerprints" to assess the process-level impacts of natural and anthropogenic activities on soils [46]. Among the three enzyme (DH, AlP, URE) activities tested, DH was the most sensitive to N fertilization history and glyphosate application rate. For example, the average application of 110 kg N ha^-1^ year^-1^ resulted in a significant decrease in enzyme activities compared to unfertilized soils. This is consistent with several studies that reported decreased DH under high N fertilizer concentrations (130 to 505 kg N ha^-1^ year^-1^), using different forms (anhydrous ammonia and urea) and applied under different climates (semi-arid sub-tropical, semi-arid and subtropical monsoon) [13,47,48]. The 100FR glyphosate application led to decreased DH activity from 15 days of incubation, regardless N fertilization history. However, the treatment with the normal field application rate of glyphosate (FR: 0.96 mg a.i ha^-1^) did not change the DH activity compared with the control. These results are consistent with Bennicelli et al. [49], who showed that both 1 and 10 mg kg-1 of glyphosate added to soils caused a decrease in DH activity that depended on the dose. However, these results are not consistent with Accinelli et al. [50], who reported stimulatory effects of glyphosate on DH activity at concentrations of 20–200 mg a.i. kg^-1^ soil. An explanation for this discrepancy was provided by Zabaloy et al. [29], who reported that changes in DH activities following the addition of high glyphosate doses (150 mg a.i kg^-1^) may depend on the history of herbicide application. However, N fertilization history did not significantly change the glyphosate application effects on DH activity, but we showed that lower DH values were measured in fertilized soil that received the high rate of glyphosate, indicating an interaction between nitrogen and glyphosate at a high glyphosate dose. By contrast, higher DH values were obtained in unfertilized soil at the normal glyphosate rate (0.96 mg kg^-1^) or in the absence of glyphosate. These results indicate that the combination of N fertilization and high dose of glyphosate application (i.e. N+\100FR) altered the DH activity, a relevant indicator of total microbial activity in soil [51]. Moreover, DH activity is used as a sensitive indicator for N flow and C use efficiency in agroecosystems [13,47] as well as in the utilization of soil O_2_ and terminal electron acceptors [52].

Similar to DH, herbicides may interact with other enzymes such as AlP or URE, through links with active protein groups. The consequences of inhibiting AlP and URE activities include the decreased transformation of urea into CO_2_ and NH_3_ and a decrease in the release of PO_4_^3-^ originating from organic P decomposition, which impacts soil fertility. The present study revealed that N fertilization induced noticeable effects on URE activity at 8 days (significant decrease with N fertilization) and 15 days (significant increase with N fertilization), while there was no effect of N fertilization history on AlP from 8 days following glyphosate application. These results are consistent with the study of Shen et al. [13], who revealed variations in URE activity over different times of sampling under high N conditions. Past studies suggested that urea added at high application rates may be used as substrate by the URE enzyme and improve its activity but also revealed that changes in soil physico-chemical properties such as humus and clay contents, pH, or salinity could inhibit its activity [53,54]. The shift observed between 8 and 15 days following glyphosate application might be induced by product-feedback inhibition of enzyme activities because of the presence of a high amount of N and P [55] induced by increased microbial growth and activity due to optimal development in the laboratory incubation (22°C and constant moisture). Indeed, we showed that both N and P contents were affected by incubation time, with increased values between 8 and 15 days.

AlP activity was impacted by the glyphosate rate, N fertilization and incubation time. One day following glyphosate application, AlP activity was higher in fertilized soils. However, high levels of mineral N may inhibit AlP activity [13,56]. AlP decreased in N0 soil from 1 to 8 days following the highest dose of glyphosate application but did not change when the herbicide was applied at the field rate (FR). However, in the N+ soil, AlP decreased from 8 to 15 days following the FR glyphosate application. The combination of the low dose of glyphosate and the N fertilization history may represent potential sources of nutrients for microorganisms and may affect the biosynthesis mechanisms of enzymes by induction or repression phenomena [57]. By contrast, glyphosate applied to unfertilized soils or glyphosate applied at the high rate did not decrease AlP activity compared with the control. Overall, the results obtained in the present study are consistent with Floch et al. [26], who revealed that URE and AlP activities yielded less insight due to their fluctuations over time and their low detection rates.

### 4.2. Glyphosate application rate modifies soil functional responses, nitrification and nutrient contents

The effects of glyphosate may be suitably assessed by testing the ability of microbial communities to degrade the 31 carbon substrates included in Biolog EcoPlates. The results obtained in the present study revealed that glyphosate has a strong dose-dependent effect on heterotrophic communities, leading to the modification of the overall degradation activity expressed by the average well color development (AWCD). At low dose (0.96 mg kg^-1^) and after 15 days of incubation, AWCD was lower in all soil samples regardless of N fertilization history. By contrast, the high dose of glyphosate (96 mg kg^-1^, 100FR) significantly increased AWCD values at 8 and mainly 15 days following application. Similarly, [34] reported that high doses (50 mg kg-1) of glyphosate increased AWCD 7 days after application. Other studies have also reported this dose-dependent response of microbial communities, also called “hormesis” [16]: at low dose, a stress response of sensitive microbial species may be observed, due to the “energy drain” and sarcosine catabolism (an intermediate in glyphosate degradation). Similarly, the decrease in AWCD in response to the FR glyphosate application may increase the residence time of soil carbon substrates, which are thus degraded more slowly. By contrast, the application of high glyphosate led to increased microbial activities from 8 days in comparison with the other soils (i.e., CK and FR) because fast-growing heterotrophic bacteria were adapted to utilize it as a C nutrient source [31,58,59]. This energy gained by microorganisms was limited by N availability, while P does not constrain microbial activity [31]. Thus, we expected a higher degradation activity in fertilized soils with glyphosate addition. Unexpectedly, N fertilization did not stimulate microbial degradation activity in the presence of glyphosate. Contrariwise, we showed that the highest glyphosate dose applied to unfertilized soil led to the highest values of functional diversity compared with fertilized soil, suggesting a better degradation of C sources as a result of a wider range of microbial functions. After 15 days following glyphosate application, the lack of a significant difference between fertilized and unfertilized soils could be due to the chemical status (pH, TOC, TN, soil C:N, CEC, NO_3_^-^, PO_4_^3-^), which was similar, suggesting that N fertilization history did not change the soil physicochemical properties before glyphosate application. Thus, glyphosate has a short-term effect on microorganisms that degrade C sources, which is greater than the long-term effect of N fertilization. On the other hand, C derived from glyphosate degradation is not the only source of interest in the herbicide since it contains other major elements potentially used as a nutrient source by microorganisms. Busse et al. [27] reported that C, N and P atoms, which comprise the glyphosate molecule, may be readily available to microorganisms that are capable to degrade these molecules.

The principal component analysis (PCA) performed (Fig 3) on the biological substrate groups revealed a higher degradation of amines in soil treated with the high glyphosate rate, especially without N fertilization. Muñoz-leoz et al., [3] suggested that SO_4_^2-^ from NPK fertilizers could reduce the microbial use of ethofumesate as an S source. Based on a similar approach, we suggest that the N contained in urea fertilizer used in the present study could reduce the microbial use of glyphosate isopropylamine salts as an N source in N-fertilized soils treated with 100FR glyphosate. Secondly, the AWCD increased and more precisely, amine degradation was positively correlated with the PO_4_^3-^ and N-NO_3_^-^ available forms, suggesting that glyphosate may be used as a P and N source by microorganisms able to achieve biological cleavage. These results are supported by previous studies, which stated that glyphosate was mainly used as a P than as an N or C source [15]. Van Eerd et al. [60] revealed that several gram-negative and gram-positive bacterial strains may use glyphosate-P, Sumalan et al. [59] stated that glyphosate could represent an additional source of C, N or P for microbial communities and Klimek-Ochab et al. [61] reported that glyphosate could be used as an N source by some fungal genera. In our study, we show that the high dose of herbicide application stimulated the activity of microbial communities (AWCD, Fig 2), which were able to degrade more amines (Fig 3).

Additionally, several studies revealed that the soil NO_3_^-^ concentration and nitrification rate could be used as suitable indicators of disturbance in N transformations [41,62]. Thereby, in our study, both higher N-NO_3_ concentrations and higher average net nitrification rates measured in the soil that received the high glyphosate treatment could be partly attributed to the deleterious effect of the herbicide on sensitive strains or to the oxidation of ammonium by surviving nitrifying bacteria (e.g., *Nitrosomonas*, *Nitrobacter*). Our results are consistent with studies of Munson et al. [63] and Vitousek and Matson [64], who showed a large increase in nitrification, N mineralization and potential nitrate losses due to reduced immobilization of N by microorganisms following herbicide application. We suggest that increased N availability is potentially involved in greater nutrient uptake by plants, thus generating a paradox in the use of herbicide application which, in parallel to represent environmental concerns (especially safety concerns), may also induce a specific N supply contributing to plant productivity.

## 5. Conclusions

From our 15-day laboratory experiment, performed to elucidate the effects of glyphosate application on soil microbial responses and nutrient content under two levels of historic N fertilization, we conclude that: 1) The normal concentration of glyphosate that is used by farmers in the field (FR, 0.96 mg kg^-1^) induced a decrease in the functional microbial activity, suggesting low degradation of carbon substrates (potentially leading to reduced losses of C stocks by mineralization); 2) when applied to unfertilized soils, the FR dose of glyphosate decreased also soil functional diversity H’ and urease activity, while only phosphatase activity was decreased by FR dose in fertilized soils; 3) a 100-fold increase in the glyphosate rate (96 mg kg^-1^) changed the bacterial community responses, resulting in increased release of nutrients from 8 to 15 days following application; 4) the application of glyphosate to soil that received nitrogen fertilization for 6 years was deleterious for enzyme activities, but this depended on the dose and the incubation time; and 5) the application of glyphosate to soils that did not receive nitrogen fertilizer increased soil NO_3_^-^ and PO_4_^3-^ contents through a probable action on the communities that degraded the herbicide. We secondly conclude that microbial and chemical responses of soils treated with the field rate of glyphosate application resemble to the response of untreated soils, and is, by against, strongly different to highly contaminated soils (100FR dose). However, our laboratory incubation being performed on plant-free soils, the extrapolation for glyphosate use in the field remains limited since herbicide application on living roots prospected soils could lead to different data. There is thus an urgent need to set up experiments assessing the field impacts of glyphosate herbicide on soil microflora.

## Supporting information

S1 Table Data matrix used for principal component analyses of Fig 3 and Fig 5. (PDF)
