# Supplementary material for: Does nitrogen fertilization history affects short-term microbial responses and chemical properties of soils submitted to different glyphosate concentrations?
Source: PLoS One. 2017 May 26;12(5):e0178342. doi: 10.1371/journal.pone.0178342 (PMC5446164; doi:10.1371/journal.pone.0178342)
Supplement: S1 Table — (PDF) [file pone.0178342.s001.pdf]

id	AMINO ACIDS	CARBOHYDRATES	CARBOXYLIC ACIDS	POLYMERS	PHOSPHATE CARBON	AMINES	DH	URE	AlP	H	AWCD	pH	CEC	Moisture	TOC	TN	soil C:N	N03	NIT	PO4	Time	Treatment
1	17,27	22,32	14,14	20,83	10,06	15,38	37,95	62,26	133,21	2,95	1,05	6,99	15,20	17,83	11,14	1,12	9,96	4,46	-1,72	41,73	Day 1	N+\CK
2	18,20	19,77	14,23	24,31	12,29	16,15	40,82	126,31	112,83	2,76	0,90	6,74	15,71	17,43	10,35	1,12	9,25	4,76	-1,42	40,83	Day 1	N+\CK
3	22,68	15,66	16,42	17,82	15,67	11,05	45,88	93,78	98,51	2,77	0,93	6,67	13,50	17,38	12,47	1,32	9,46	5,01	-1,17	44,03	Day 1	N+\CK
4	23,01	16,41	16,58	18,18	10,89	14,93	37,30	86,46	142,79	2,95	0,91	6,99	12,95	18,98	11,33	1,14	9,96	5,35	-0,83	47,91	Day 1	N+\CK
5	24,62	18,99	13,78	13,49	7,53	18,10	41,01	78,19	142,26	2,39	0,82	6,87	13,90	17,76	11,13	1,16	9,60	8,31	-1,24	45,96	Day 1	N+\CK
6	22,32	26,48	16,12	15,98	13,46	11,31	40,94	99,88	141,64	2,86	0,83	6,58	13,96	17,96	11,60	1,18	9,87	5,12	-1,06	45,43	Day 1	N+\CK
7	19,30	18,36	14,20	24,00	10,30	13,84	40,62	121,69	139,37	2,83	0,87	6,96	11,93	20,58	11,40	1,17	9,74	5,92	-0,27	44,53	Day 1	N+\FR
8	18,03	20,10	22,11	18,65	4,41	16,69	36,48	102,92	105,25	2,79	0,92	6,93	15,42	17,75	11,53	1,23	9,40	4,99	-1,19	46,83	Day 1	N+\FR
9	18,53	19,77	13,68	25,05	6,14	16,84	40,95	133,25	122,94	2,71	1,04	6,87	11,61	18,84	13,11	1,20	10,96	6,70	0,52	46,91	Day 1	N+\FR
10	24,30	21,09	17,01	16,10	5,85	15,65	45,31	133,56	101,56	2,75	0,85	6,69	14,24	18,15	12,72	1,36	9,35	6,48	0,30	46,59	Day 1	N+\FR
11	17,92	17,17	17,60	22,60	6,54	18,17	37,12	105,46	147,47	2,90	0,93	6,99	11,05	18,04	11,71	1,19	9,85	6,60	0,42	45,34	Day 1	N+\FR
12	16,61	16,95	21,12	18,57	8,32	18,44	48,49	85,79	94,91	2,90	0,97	7,03	14,09	18,26	11,53	1,16	9,91	7,03	0,85	46,23	Day 1	N+\FR
13	19,39	14,70	15,74	22,28	12,90	15,01	32,93	94,54	112,84	2,77	0,94	6,95	11,90	18,30	12,90	1,58	8,18	8,41	2,23	49,52	Day 1	N+\100FR
14	22,15	14,53	20,50	16,95	8,48	10,66	42,45	111,15	107,44	3,09	0,89	6,93	12,34	17,84	11,55	1,20	9,59	7,54	1,36	52,01	Day 1	N+\100FR
15	20,30	18,55	16,81	22,77	7,92	13,65	32,40	104,06	96,71	2,85	0,80	6,80	10,38	18,44	11,51	1,16	9,94	8,23	2,05	53,61	Day 1	N+\100FR
16	21,03	21,71	19,71	19,23	8,80	9,52	38,37	167,69	108,52	2,76	0,68	6,95	14,98	18,01	11,56	1,19	9,72	6,72	0,54	57,19	Day 1	N+\100FR
17	19,11	17,41	19,15	14,90	13,76	15,68	39,96	141,97	103,10	2,82	0,90	6,97	13,32	18,66	11,76	1,21	9,69	6,21	0,03	48,70	Day 1	N+\100FR
18	26,41	20,11	19,55	17,20	8,38	10,43	35,46	109,50	90,35	2,34	0,78	6,97	9,92	18,45	11,76	1,14	10,30	6,17	-0,01	47,81	Day 1	N+\100FR
19	18,36	19,10	15,04	21,26	13,17	13,07	50,71	159,29	91,09	3,13	1,16	6,66	12,85	18,40	12,65	1,28	9,89	5,76	-0,24	48,00	Day 1	N0\CK
20	18,71	20,21	15,51	19,50	11,68	14,39	50,27	95,73	71,84	3,00	1,08	6,93	12,69	18,17	11,82	1,16	10,17	5,03	-0,98	52,99	Day 1	N0\CK
21	17,95	19,84	15,53	19,96	13,86	12,87	46,55	102,68	71,32	2,99	1,00	6,92	11,02	21,18	11,86	1,13	10,49	5,60	-0,41	51,13	Day 1	N0\CK
22	16,02	19,31	15,53	18,98	11,99	18,17	42,32	112,09	79,36	3,09	1,06	6,96	13,24	20,86	11,90	1,16	10,25	5,61	-0,40	44,49	Day 1	N0\CK
23	15,53	20,64	18,07	17,31	13,19	15,26	47,84	113,42	103,05	2,94	1,00	6,55	12,01	17,90	12,13	1,16	10,45	5,20	-0,80	45,92	Day 1	N0\CK
24	17,40	20,11	18,59	22,57	11,68	9,65	48,79	102,26	87,13	3,11	0,96	6,92	14,17	18,15	12,77	1,20	10,65	5,95	-0,05	46,89	Day 1	N0\CK
25	16,70	21,06	14,61	24,59	10,85	12,20	50,73	126,83	72,77	3,02	0,91	6,63	13,55	18,65	12,00	1,14	10,53	9,09	0,99	42,73	Day 1	N0\FR
26	19,56	21,47	13,94	21,02	9,85	14,16	56,87	81,94	109,86	3,00	0,99	6,95	12,97	18,58	12,08	1,16	10,39	7,10	1,09	41,41	Day 1	N0\FR
27	18,74	22,71	15,80	18,84	12,40	11,52	50,86	115,41	85,32	3,07	1,15	7,10	11,44	18,42	14,82	1,17	12,64	7,41	1,40	44,32	Day 1	N0\FR
28	18,43	20,05	17,62	24,36	5,63	13,91	50,97	59,67	100,29	2,95	1,02	6,90	13,80	18,15	12,02	1,20	10,05	5,97	-0,04	45,35	Day 1	N0\FR
29	17,68	18,23	15,41	21,90	13,33	13,45	52,28	91,73	104,20	3,00	1,11	6,63	12,33	19,79	12,08	1,25	9,64	7,73	1,73	46,22	Day 1	N0\FR
30	19,27	18,82	16,42	20,83	10,64	14,02	52,07	95,88	115,93	2,70	0,84	7,00	11,75	18,38	13,25	1,28	10,38	6,79	0,78	46,80	Day 1	N0\FR
31	16,34	20,62	15,82	21,60	9,08	16,53	45,74	103,46	55,08	2,99	1,01	6,99	10,79	17,61	13,85	1,62	8,57	7,02	1,01	51,78	Day 1	N0\100FR
32	18,80	18,33	15,88	18,10	12,82	16,07	50,55	62,10	58,20	2,75	0,78	6,81	11,07	18,29	12,06	1,21	9,93	7,83	1,82	53,51	Day 1	N0\100FR
33	16,34	16,77	15,29	21,94	10,96	18,70	45,28	92,21	69,82	2,88	0,78	6,63	10,75	18,64	12,08	1,23	9,85	7,73	1,72	53,53	Day 1	N0\100FR
34	19,85	19,21	21,88	21,12	9,90	8,04	48,22	113,25	45,72	2,43	0,96	6,93	9,00	18,55	13,28	1,24	10,73	7,63	1,62	51,45	Day 1	N0\100FR
35	15,58	22,10	15,18	21,02	10,60	15,52	42,77	90,45	56,65	3,07	1,12	6,83	11,12	18,49	13,62	1,20	11,38	11,28	1,94	50,26	Day 1	N0\100FR
36	16,74	15,93	17,65	24,21	10,43	15,05	44,84	81,23	51,21	2,79	0,87	6,97	9,90	18,45	11,66	1,15	10,11	9,51	3,50	50,40	Day 1	N0\100FR
37	22,28	19,78	16,77	21,19	4,30	15,68	30,05	85,80	122,87	2,74	0,84	6,98	11,08	17,07	12,04	1,20	10,00	5,95	-0,03	40,24	Day 2	N+\CK
38	29,37	17,73	19,79	16,84	2,82	13,45	54,71	85,80	104,37	2,65	0,71	6,86	10,59	16,97	12,05	1,36	9,91	8,01	0,23	40,18	Day 2	N+\CK
39	23,71	14,82	17,53	18,33	3,95	9,54	26,36	86,20	108,14	2,88	0,87	6,82	11,18	17,70	12,10	1,26	9,58	7,61	0,18	38,12	Day 2	N+\CK
40	24,46	16,15	20,47	20,73	6,03	12,16	55,73	85,85	100,45	2,72	0,77	7,11	11,31	17,93	12,17	1,24	9,82	7,35	0,15	40,25	Day 2	N+\CK
41	24,14	13,96	20,74	23,68	4,34	13,14	29,14	88,82	129,41	2,61	0,53	6,79	11,08	22,12	12,65	1,22	10,40	6,61	0,05	46,16	Day 2	N+\CK
42	24,76	14,72	18,50	25,14	2,28	14,60	26,84	82,33	103,74	2,76	0,81	6,79	11,25	18,24	11,29	1,16	9,76	6,69	0,06	40,76	Day 2	N+\CK
43	20,93	19,04	17,36	20,02	5,90	16,76	37,55	82,33	87,98	2,93	0,97	7,03	10,56	18,28	11,98	1,23	9,73	7,62	0,18	41,06	Day 2	N+\FR
44	17,77	16,70	15,79	17,91	12,51	19,32	47,18	87,16	71,90	3,04	1,07	6,78	11,18	17,06	15,83	1,25	12,68	7,19	0,13	48,64	Day 2	N+\FR
45	22,95	20,04	17,80	21,95	1,25	16,01	38,85	84,94	59,52	2,91	1,04	6,87	10,67	18,30	12,43	1,25	9,94	10,08	0,49	40,24	Day 2	N+\FR
46	20,86	18,58	18,76	19,10	5,53	17,18	61,57	78,57	56,53	2,95	0,93	6,88	9,39	18,13	11,54	1,17	9,89	4,35	-0,23	39,71	Day 2	N+\FR
47	23,53	23,29	16,97	16,13	3,73	16,36	40,56	83,95	48,53	2,82	0,93	6,90	11,23	17,05	12,21	1,25	9,80	6,87	0,09	43,35	Day 2	N+\FR
48	19,51	16,59	17,48	24,98	6,49	14,94	49,51	85,01	99,63	2,91	0,89	6,82	10,98	18,12	12,91	1,35	9,55	6,86	0,08	38,29	Day 2	N+\FR
49	23,46	13,21	20,56	19,67	0,51	22,59	40,66	82,13	111,82	2,79	1,07	6,89	10,91	17,20	12,14	1,25	9,73	16,49	1,29	43,30	Day 2	N+\100FR
50	22,54	9,78	19,87	20,73	0,63	26,45	46,13	76,89	116,18	2,76	1,07	6,93	10,65	17,29	19,67	1,31	15,00	17,46	1,41	44,46	Day 2	N+\100FR
51	23,25	10,84	19,65	20,54	0,82	25,06	25,23	72,10	116,49	2,80	1,05	6,90	10,18	17,41	11,87	1,13	10,47	19,22	1,63	46,08	Day 2	N+\100FR
52	23,02	8,10	20,32	21,33	1,05	26,18	31,80	72,23	103,91	2,78	1,03	7,00	11,51	17,18	12,94	1,22	10,60	22,72	2,07	42,52	Day 2	N+\100FR
53	22,13	11,88	18,36	23,20	1,10	23,33	36,28	74,87	108,59	2,90	1,17	6,91	10,90	17,18	12,02	1,15	10,44	18,38	1,53	44,54	Day 2	N+\100FR
54	25,09	11,21	19,16	17,79	0,82	26,75	26,57	82,54	119,96	2,77	0,90	6,93	11,31	18,02	13,26	1,19	11,12	14,56	1,05	45,53	Day 2	N+\100FR
55	19,49	19,76	20,27	19,80	3,45	17,22	59,02	96,45	122,98	2,91	0,99	6,96	10,99	18,46	12,66	1,19	10,67	10,49	0,56	39,71	Day 2	N0\CK
56	23,53	18,84	18,45	17,63	8,37	15,20	46,58	89,26	108,14	2,81	0,87	6,99	10,55	17,83	12,32	1,20	10,27	6,56	0,07	43,80	Day 2	N0\CK
57	17,57	23,08	16,89	23,97	6,26	12,23	54,99	94,30	117,90	2,96	1,06	6,84	9,66	11,29	12,38	1,17	10,56	6,39	0,05	41,06	Day 2	N0\CK
58	22,37	20,23	23,04	18,96	2,03	13,36	60,25	93,14	98,73	2,81	0,83	6,91	10,55	18,48	11,93	1,15	10,41	10,88	0,61	41,61	Day 2	N0\CK
59	23,52	17,62	19,39	27,25	1,21	11,01	52,74	91,48	106,51	2,84	0,78	6,85	10,73	17,57	12,43	1,14	10,90	7,77	0,22	47,48	Day 2	N0\CK
60	16,82	23,77	20,41	18,39	7,51	13,10	39,19	96,38	82,71	2,90	0,94	6,81	10,80	18,57	15,94	1,20	13,32	7,28	0,16	49,57	Day 2	N0\CK
61	23,73	13,63	22,42	20,01	6,86	13,35	49,46	94,38	93,08	2,74	0,80	6,92	12,18	17,87	12,38	1,18	10,48	5,46	-0,07	48,47	Day 2	N0\FR
62	17,83	17,25	19,21	27,82	1,56	16,33	56,56	92,08	104,39	2,75	0,72	6,80	11,18	18,91	12,39	1,14	10,85	8,23	0,28	44,92	Day 2	N0\FR
63	23,37	22,98	17,37	21,12	2,08	13,08	40,13	90,06	87,55	2,86	0,87	6,78	9,91	18,75	12,38	1,19	10,41	7,38	0,17	41,98	Day 2	N0\FR
64	19,84	16,62	16,66	20,70	10,89	15,29	54,20	91,55	139,11	2,93	0,91	6,88	11,36	17,99	13,75	1,36	10,13	9,45	0,43	44,53	Day 2	N0\FR
65	23,99	19,53	18,12	21,64	1,07	15,65	36,15	93,07	140,99	2,67	0,67	6,90	11,22	18,25	13,10	1,24	10,54	9,81	0,47	43,36	Day 2	N0\FR
66	18,26	19,73	19,29	17,42	11,98	13,32	46,30	93,39	140,64	2,90	0,81	6,99	11,05	19,12	12,45	1,23	10,10	6,87	0,11	42,04	Day 2	N0\FR
67	21,18	11,37	18,81	18,66	1,40	28,59	51,78	96,75	98,71	2,91	1,22	6,77	11,93	17,93	11,66	1,23	9,50	15,54	1,19	43,57	Day 2	N0\100FR
68	20,42	14,91	18,40	14,97	0,66	30,63	26,15	117,55	81,02	2,87	1,29	6,83	10,84	19,02	13,17	1,26	10,47	21,48	1,93	44,19	Day 2	N0\100FR
69	21,72	16,47	18,72	14,94	4,50	23,66	41,03	121,44	94,20	2,93	1,03	6,94	13,10	18,99	12,64	1,17	10,84	20,42	1,80	45,37	Day 2	N0\100FR
70	20,93	13,77	18,53	14,27	5,16	27,33	47,65	105,81	62,82	2,87	1,20	6,91	10,43	18,53	11,58	1,13	10,24	18,70	1,59	52,26	Day 2	N0\100FR
71	19,44	17,14	17,25	17,24	4,67	24,26	43,58	105,03	79,81	2,91	1,30	6,82	11,27	18,37	13,21	1,21	10,89	18,30	1,54	49,02	Day 2	N0\100FR
72	21,17	14,25	18,92	17,31	1,03	27,31	36,67	102,54	69,56	2,92	1,22	6,72	10,62	18,14	12,35	1,21	10,19	16,60	1,32	51,63	Day 2	N0\100FR
73	22,01	13,77	19,13	22,76	8,52	13,81	53,56	125,64	69,04	3,01	0,72	6,95	10,73	16,62	12,60	1,46	8,61	9,05	0,19	46,00	Day 3	N+\CK
74	22,26	22,12	20,97	16,24	4,83	13,58	46,40	125,64	82,62	2,84	0,88	7,05	12,07	17,19	12,82	1,31	9,78	7,87	0,11	43,81	Day 3	N+\CK
75	22,77	13,99	17,88	19,53	2,57	23,25	67,72	125,64	74,02	2,72	0,80	7,06	11,81	16,37	12,95	1,31	9,90	11,58	0,36	42,11	Day 3	N+\CK
76	27,69	15,24	21,86	22,16	1,87	11,18	53,70	125,64	79,08	2,72	0,75	6,86	10,36	18,01	12,48	1,31	9,53	7,25	0,07	44,56	Day 3	N+\CK
77	20,65	15,47	25,44	20,20	1,38	16,86	58,72	128,16	75,54	2,74	0,69	7,00	10,63	18,34	12,76	1,29	9,87	7,12	0,06	46,33	Day 3	N+\CK
78	24,28	15,88	20,92	18,53	1,32	19,08	42,87	123,12	83,71	2,61	0,89	6,79	12,10	16,75	13,40	1,33	10,10	6,54	0,02	45,56	Day 3	N+\CK
79	24,08	13,91	17,26	19,82	1,45	15,46	57,07	130,42	46,13	2,77	0,67	6,91	9,91	18,33	12,61	1,45	8,71	8,43	0,15	42,76	Day 3	N+\FR
80	26,21	8,10	18,25	27,04	0,66	19,73	45,24	128,87	44,43	2,65	0,38	6,93	10,47	16,82	11,80	1,42	8,31	8,99	0,19	42,64	Day 3	N+\FR
81	25,01	15,80	14,81	25,86	3,76	14,76	49,06	127,16	45,62	2,56	0,56	6,92	11,75	18,02	13,78	1,31	10,51	8,91	0,18	43,92	Day 3	N+\FR
82	24,91	19,35	17,84	28,89	3,44	16,67	52,95	124,09	47,55	2,43	0,55	6,95	11,91	18,30	13,91	1,41	9,83	7,78	0,11	44,03	Day 3	N+\FR
83	26,64	14,83	18,15	22,23	4,14	14,01	52,24	124,91	60,21	2,32	0,52	6,92	11,75	17,59	13,54	1,42	9,53	7,72	0,10	44,22	Day 3	N+\FR
84	23,70	11,58	17,72	22,68	7,18	26,69	45,94	121,37	65,54	2,69	0,51	6,93	11,16	17,72	12,37	1,31	9,44	8,19	0,13	41,18	Day 3	N+\FR
85	23,89	11,00	19,01	16,01	2,15	27,93	45,69	121,38	58,59	2,61	1,01	6,89	11,72	17,29	13,48	1,36	9,89	22,16	1,06	46,11	Day 3	N+\100FR
86	24,34	6,09	20,27	17,74	4,44	27,13	41,86	113,81	76,51	2,40	0,93	6,91	12,54	17,36	12,76	1,30	9,81	18,29	0,81	44,31	Day 3	N+\100FR
87	24,98	9,29	18,49	20,75	0,72	25,78	46,78	113,38	78,15	2,79	0,92	6,91	12,07	17,91	12,11	1,26	9,60	18,53	0,82	45,45	Day 3	N+\100FR
88	24,35	13,33	16,52	14,49	5,76	25,55	36,25	117,27	88,96	2,64	1,07	7,00	11,06	17,41	11,97	1,24	9,62	16,86	0,71	50,62	Day 3	N+\100FR
89	20,13	11,38	17,09	16,98	10,90	23,51	37,31	115,32	63,58	2,72	1,17	6,93	11,20	17,86	12,51	1,31	9,55	14,60	0,56	46,64	Day 3	N+\100FR
90	24,12	11,59	18,78	16,09	4,80	29,41	43,01	113,04	71,64	2,94	1,05	6,84	10,38	18,37	13,60	1,37	9,94	21,27	1,01	48,75	Day 3	N+\100FR
91	22,89	17,78	19,41	18,09	4,39	17,44	56,43	85,56	80,15	2,73	0,77	6,90	10,90	18,05	13,36	1,37	9,77	8,58	0,17	45,71	Day 3	N0\CK
92	19,89	18,17	18,08	23,54	3,61	16,71	57,70	79,17	70,10	2,82	0,81	6,86	11,75	17,42	13,51	1,39	9,71	10,57	0,30	47,38	Day 3	N0\CK
93	21,34	12,73	23,74	18,59	8,16	15,44	49,96	79,65	76,52	2,78	0,78	7,00	9,32	18,21	12,49	1,34	9,32	10,54	0,30	45,32	Day 3	N0\CK
94	20,54	18,39	16,10	20,08	8,15	16,74	69,45	77,60	65,96	2,79	0,74	7,01	9,05	17,72	13,31	1,33	10,03	8,38	0,16	45,47	Day 3	N0\CK
95	20,53	15,88	15,42	20,22	10,78	17,16	57,22	84,70	73,18	2,68	0,72	7,03	10,80	17,34	14,36	1,55	9,26	12,25	0,42	46,57	Day 3	N0\CK
96	23,99	18,49	17,73	23,88	0,47	15,45	53,90	76,83	73,18	2,63	0,70	6,97	11,05	18,23	12,55	1,29	9,72	10,61	0,31	45,42	Day 3	N0\CK
97	25,15	16,12	26,14	23,65	4,70	14,57	54,75	83,43	92,95	1,92	0,50	6,91	11,70	17,94	12,54	1,35	9,31	10,06	0,27	45,95	Day 3	N0\FR
98	24,29	18,00	24,02	20,10	4,67	15,37	48,08	83,28	90,06	2,38	0,36	6,94	10,21	17,98	13,24	1,38	9,57	10,53	0,30	46,72	Day 3	N0\FR
99	31,38	19,27	24,00	22,81	4,67	14,57	55,82	74,96	89,26	2,02	0,30	7,03	10,05	18,46	14,80	1,50	9,87	10,39	0,29	45,78	Day 3	N0\FR
100	22,21	13,56	18,16	25,10	5,39	15,58	66,61	78,40	80,24	2,59	0,58	6,99	10,89	17,49	13,69	1,42	9,64	9,23	0,21	45,98	Day 3	N0\FR
101	26,36	15,83	16,81	22,06	6,06	12,87	50,67	76,30	80,80	2,62	0,63	7,03	9,99	18,67	14,23	1,44	9,91	9,81	0,25	48,00	Day 3	N0\FR
102	21,62	13,96	19,28	28,18	2,52	14,45	57,44	82,86	63,51	2,60	0,62	6,98	9,88	18,16	13,19	1,43	9,24	10,30	0,29	48,06	Day 3	N0\FR
103	20,76	12,75	15,25	20,28	5,37	25,59	38,57	81,90	97,25	2,83	1,08	6,98	11,01	16,80	12,86	1,48	8,66	20,72	0,98	49,39	Day 3	N0\100FR
104	17,79	12,85	16,25	23,62	7,32	22,17	42,70	84,78	84,69	3,01	1,20	6,90	11,45	17,79	14,35	1,53	9,37	24,82	1,25	51,07	Day 3	N0\100FR
105	20,85	17,09	18,30	18,47	0,92	24,37	46,35	94,94	70,58	2,83	1,08	7,01	9,79	18,36	13,74	1,55	8,89	26,47	1,36	51,54	Day 3	N0\100FR
106	20,91	15,88	17,09	19,43	1,73	24,96	41,43	96,73	88,61	2,88	1,17	6,90	9,45	17,84	13,72	1,31	10,45	18,60	0,84	58,99	Day 3	N0\100FR
107	22,70	12,62	17,18	18,21	4,52	24,77	45,56	84,95	90,17	2,99	1,15	7,01	11,48	17,75	12,50	1,29	9,71	20,91	0,99	51,35	Day 3	N0\100FR
108	24,28	15,01	17,84	19,57	0,96	22,34	55,75	102,67	76,05	2,85	0,99	7,01	10,62	17,01	12,96	1,47	8,84	22,13	1,07	50,50	Day 3	N0\100FR
